# Supplementary material for: Self-reported uptake of STI testing services among adolescents and young people aged 15–24 years: Findings from the Yathu Yathu cluster randomized trial in Lusaka, Zambia
Source: PLOS Glob Public Health. 2024 Mar 4;4(3):e0002491. doi: 10.1371/journal.pgph.0002491 (PMC10911628; doi:10.1371/journal.pgph.0002491)
Supplement: Attachment — Submitted filename: Response_reviewer_comments_revised.docx [file pgph.0002491.s003.docx]

**Reviewer #1:**

Relatively high numbers of AYP presenting for syndromic STI screening was likely a result of the incentivisation process through the PPC. This influence might have been considered further in the Discussion section, particularly when the authors argued for structural interventions to address drivers of vulnerability to STIs amongst AYP.

Thank you for this point. We agree that the incentives likely contributed to the high numbers of AYP attending hubs and have made this clear in the third paragraph of the discussion by adding: *The availability of incentives likely contributed to the relatively high number of AYP who opted for syndromic screening at the Yathu Yathu hubs; future iterations of the model should continue to offer incentives, including products to enhance dental and menstrual health.*

Similarly, the association between household poverty and STI exposure amongst AGYW might have been strengthened by the inclusion of evidence for what the authors themselves describe as 'a tenuous link'.

Thank you for raising this point, we agree we should have included literature supporting this relationship. We have now revised this paragraph to read: *Although a tenuous link in our study, AGYW who experience food insecurity and have less household-level wealth may be more likely to engage in selling sex, to know their risk of, and therefore to test for, STIs. An analysis of a nationally representative survey of adults aged 15-44 in the USA demonstrated an association between food insecurity and STI risk indicators (including previous diagnosis of chlamydia or gonorrhea)(36). Similarly, a survey of university students in South Africa found an association between food insecurity and engaging in transactional sex for “money” or “to meet basic needs”(37).*

**Reviewer #2: General comments**

1. It is not clear what it is that was offered to the adolescents and young people in the control arm, besides the prevention points cards that seemed to be offered to all participants. A clear description of the services in the control arm, which the participants accessed, preferably as part of routine care, should be provided.

Thank you for indicating that this was not clear. In response, on page 6, paragraph 4, we have included the sentence: *Each community had one public health facility offering STI testing and treatment services and youth-friendly corners; as such, these were the services available in the control arm. An information desk was established at both facilities to welcome AYP and allow them to accrue points for services accessed at the health facility.*

2. The authors indicate the following study outcomes: “the percentage of AYP self-reporting ever testing for an STI, other than HIV, among AYP reporting ever having had sex, and the percentage of AYP self-reporting recently (last 12 months) testing for an STI among AYP reporting sex in the last 12 months”. I have 2 comments here:
a) Ever testing for STI could mean any testing done before the Yathu Yathu services became available in August 2019. How did the authors ensure that any testing referred to here was a result of the Yathu Yathu intervention? I thought that some of the variables used to assess the association with ever STI testing should have included exposure to the Yathu Yathu intervention, if the purpose was to link any STI testing to the intervention effect.

Thank you for this comment. Our analysis comparing the intervention to the control arm aimed to estimate whether the intervention had an impact on ever- or recent-testing (in the last 12 months) for an STI. We included both outcomes as we anticipated that many AYP would not have tested for an STI prior to the availability of Yathu Yathu; however, our assumption could have been incorrect as the Reviewer points out – we therefore also included STI testing in the last 12 months to account for an effect that could be influenced by the Yathu Yathu intervention. This analysis found no significant difference across the two trial arms, other than for men as described in the results. As there was no effect of the intervention in this analysis, we did not look at the effect of Yathu Yathu in the risk factor analysis. However, we did control for intervention arm in the analysis for men, considering there was evidence of an effect among men.

b) The authors collected data from two sources: the process data from the prevention point cards and the survey. How were the two data sources linked to ensure that the data were referring to the same person? For instance, how were the data collected through PPCs linked to the survey data to indicate that those who reported recent STI testing (in the last 12 months) had had access to STI services via Yathu Yathu?

Although the PPC data could be linked to the survey data using the unique PPC number, we did not link these data for this analysis. The reason for this is that we aimed to use the PPC data to describe the process of implementing the services, as such focussing on use of syndromic STI screening services. We used the endline survey data to estimate the population-level impact of Yathu Yathu on STI testing and explore factors associated with ever STI testing. All AYP participating in the survey had been provided a PPC during the census, but they may not have accessed any services from the Yathu Yathu hubs. If we had limited our analysis of the endline survey to AYP who had accessed services at the hubs, this would likely have biased our results on the impact of Yathu Yathu at population-level.

c) In the main results, a logistic regression analysis is conducted for ever-STI testing. But the authors mention two different outcomes, including STI testing in the last 12 months. However, I did not any regression analysis for recent STI testing which to me would have been a more close measure of the impact of the intervention than ever-testing.

Thank you for this comment. On page 8, paragraph 2, we mention that we opted not to look at factors associated with STI testing in the last 12 months because the implementation of Yathu Yathu was affected by the COVID-19 control measures: *As COVID-19 control measures likely affected STI service delivery at the health facility in the year prior to the survey, we did not explore factors associated with recent STI-testing.* We therefore opted to look at ever STI testing to ensure we could capture the impact of Yathu Yathu when it was first implemented and not affected by COVID-19 control measures.

3. Since the Yathu Yathu was implemented as a CRT, the authors should include a CONSORT statement (extension to cluster randomized trials) as required in the reporting of such trials.

Thank you for this comment. We have included the CONSORT statement checklist and mentioned, in the methods, that we have reported our findings in line with the CONSORT guidelines. We report this under Methods: W*e report analyses of the CRT in line with the CONSORT extension for Cluster Randomised Trials (Supplementary file 1). (16)*

4. In general, I did not see how the data from PPCs (as one of the two data sources) were used to inform the reporting of results obtained through the endline survey (the second data source). For instance, I think that, based on data from PPCs, some 6.2% (n=679) of the participants reported any STI symptoms. Then, based on the results from the endline survey, unless I missed it, some 6.6% (n=23) reported ever having any STI symptoms. So, how did the authors use data from the PPCs and that from the endline survey during the reporting on the impact of Yathu Yathu on STI screening and testing among AYP? I think a response to these questions can help to justify if the two data sources were necessary.

The PPC data were used to describe the process of service delivery, and can be viewed as routine monitoring data. We have made this clearer on page 6, the final paragraph, in the sentence: *The first data source was process data on services accessed by AYP attending Yathu Yathu hubs, which were routinely collected using the PPC.* The endline survey was conducted in a random sample of AYP who had received a PPC, but may not have accessed Yathu Yathu services. This survey was conducted to estimate the population-level impact of the intervention. As such, we do not combine the data but rather use one source to describe the process of delivery and who was reached, and the second to estimate impact and understand who has ever STI tested.

Specific comments

1. Were the study communities divided into 10 or 20 zones? In the abstract, reference is made to 10 zones. However, on page 5 (study location and population), a total of 20 zones is used. Please clarify.

Thank you for this comment. Each community was divided into 10 zones. We have made this clearer in the abstract by stating: *Two communities in Lusaka were divided into 10 zones each (20 zones in total);*

2. In the introduction section, the authors make a profound case for increases in STIs among adolescents aged 10-14 years, despite a general decrease in STIs among adolescents and young people (AYP) aged 10-24 years. However, their study recruited those aged 15-24 years. Why didn’t enroll the population group that is most at risk?

This is a secondary analysis of data collected for a CRT to estimate the impact of Yathu Yathu on knowledge of HIV status among 15 to 24 year olds. As such, at the time of designing the study we did not consider enrolling younger age groups.

3. How did the authors define ‘STI screening’? How was it done?

As described in the section on the Yathu Yathu intervention, STI screening at the hubs was syndromic. If an AYP asked for syndromic screening, they were asked whether they were experiencing specific symptoms and referred to the nearest health facility if they were experiencing any symptoms.

4. How soon did the AYP go for STI testing after being screened with STI symptoms? Did the authors see any differences between arms? Did the authors find out if the AYP who tested positive for STIs (4.3% for syphilis and 0.9% for gonorrhea) sought treatment?

The AYP were referred to the health facility if they experienced any STI symptoms but they were not followed up. If they were diagnosed with an STI at the health facility and initiated treatment, they could return to the hubs and report this to the peer support workers. We have included details of this in paragraph 3 of the methods: *If AYP were referred to the nearest health facility, tested positive for an STI and initiated treatment for this STI, they could gain 250 points for initiating treatment. Yathu Yathu services were available from September 2019.*

5. In the results section, the authors write: “At their first STI symptom screen, 7.7% (n=563) of AYP reported experiencing any STI symptom…” I am not sure I understand the denominator for this percentage, given that there is already a 6.2% (n=679) of AYP reporting symptoms. To improve this statement, the authors may revise thus: ‘… At their first STI symptom screening, 7.7% (n=563) of AYP who xxxx, reported experiencing any STI symptom”. This can help to qualify the 7.7% as a percentage derived from a defined sub-population.

Thank you for pointing out that this was not clear. The first percentage is the percentage of AYP who reported any symptom at any STI syndromic screening visit. We then focussed specifically on the first screening visit (as we assumed a higher percentage would report symptoms at a first screen than subsequent screening visits); as such, the second percentage is the percentage of AYP who reported any symptoms at their *first* syndromic STI screening visit. We have made this clear by revising the following sentence on page 9, second paragraph: *Among these AYP, 39.6% (n=2898/7310) were screened more than once and 6.2% (n=679) reported any STI symptoms at any visit (10.8%, n=511 females and 6.5%, n=168 males).* And in the third paragraph have revised the sentence to include the denominator: *At their first STI symptom screen, 7.7% (n=563/7310) of AYP reported experiencing any STI symptom (8.8%, n=419, of females and 5.6%, n=144, of males)…*

6. In the results section, I suggest that the authors begin by describing the population studied (whose characteristics are shared in Table 1). At the moment, the results section begins with “Syndromic STI screening at Yathu Yathu services” – before the readers get to know the characteristics of the population studied.

We prefer to keep the results as presented; this is because the first data source describes access to (and so delivery of) the services. Only after this do we present results from the endline survey and therefore describe the characteristics of the individuals who consented to complete the survey. We consider this order to be clearer as the reader can then first understand service uptake and then impact at population level.

7. Given differences in sexual behavior between adolescent boys and adolescent girls, I would present Table 1 stratified by sex (across study arms) so that we can see these differences between adolescent boys and girls by study arm across the different background characteristics.

As the objective of the study is not to compare the characteristics of the population by sex, we prefer to keep Table 1 as an overall Table. Furthermore, in Tables 3 and 4 we present the characteristics of the study population stratified by sex. If we were to stratify Table 1 this would repeat information provided in later tables.

8. In table 1, the authors should describe what the “wealth quintile” levels 1-5 stand for. A descriptor should be included. Besides, the term “wealth quintile” is used for a 3-tier arrangement. I don’t think it would be appropriate to refer to a 5-tier level with a “quintile”.

Thank you for pointing out that we need to include a descriptor, we have now included this in the tables. We have, however, not changed the term wealth quintile, as a quintile is five groups and our wealth variable is indeed divided into five groups rather than three.

9. On page 9, the authors write, “Overall, 1989 AYP participated in the endline survey, among whom 75.7% (n=1505) reported ever having had sex and 1501 had complete data on testing for STIs”. It would be nice if the authors included a CONSORT diagram that displays how the final numbers in each arm that were used in the analysis (1501) were derived.

Thank you for this suggestion, we have included a flowchart that illustrates the final number of survey participants and participants whose data were included in the analyses reported.

10. On page 11, the authors write, “Overall, 23.3% (n=350/1501) of AYP who ever had sex reported ever testing for an STI, other than HIV, and 13.5% (n=148/1099) of AYP who reported sex in the last 12 months also reported testing for an STI in the last 12 months”. This statement mixes two different results (i.e. ever STI testing in those who had ever had sex separately from those who had sex in the past 12 months, yet those who had sex in the past 12 months are part of those who have ever had sex) making interpretation of the statement a little difficult. I suggest that the authors separate it into two separate sentences:
a) Overall, 23.3% (350/1501) who ever had sex reported ever testing for an STI other than HIV; of these, xxx% (n=xxx) were sexually active in the past 12 months of whom xxx% (xxx/yyy) reported that they tested for STIs in the past 12 months.

b) Of 1,099 AYP who reported sex in the past 12 months, xxx% (xxx/yyy) reported having any STI symptoms; of these, xxx% (xxx/yyy) reported that they tested for STIs in the past 12 months; or something in this regard.

Thank you for pointing out that this sentence was not clear. In response to this concern, we have rewritten this sentence as two sentences (page 11, 1^st^ paragraph): *Overall, 23.3% (n=350/1501) of AYP who ever had sex reported ever testing for an STI, other than HIV. Restricting to AYP who reported ever having sex in the last 12 months, 13.5% (n=148/1099) reported testing for an STI in the last 12 months.*

11. Page 14 begins, “Among those who ever tested for an STI, 6.6% (n=23) reported ever being diagnosed with an STI; five individuals did not know which STI they tested positively for.” It is not clear to me – which denominator was used to derive the reported 6.6%. I suggest that whenever sub-group analyses are reported, the authors should describe the population sub-group referred to and provide a denominator to help in qualifying the percentage.

Thank you for pointing this out, we agree we should have included the denominator. We have revised this sentence to read: *Among AYP who ever tested for an STI, 6.6% (n=23/350) reported ever being diagnosed with an STI; five individuals did not know which STI they tested positively for.*

12. About Table 3:
a) How did the authors ensure that the ever-STI testing was associated with the Yathu Yathu intervention? Why didn’t the authors determine the factors associated with recent STI testing, which was more likely to be associated with the intervention, given the timing?

b) Why did the authors opt to use odds ratios when the prevalence of the outcome was higher than 10%?

c) It is not clear if the results presented have been adjusted for clustering effect and exposure to the intervention.

d) These comments also apply to Table 4.

Thank you for these comments on Tables 3 and 4.

a. As per our methods and a response to an earlier comment, considering the potential implications of COVID-19 control measures on access to STI testing services at the nearest health facilities we thought it prudent to use ever STI testing as our outcome in our risk factor analysis. We also saw no intervention effect in our CRT analysis, other than amongst men, and therefore did not explore the association between residing in a Yathu Yathu zone and the outcome.
b. We used the OR as this is standard with logistic regression. None of our OR are very large, so we considered it appropriate to present OR rather than convert these to PR.
c. As described in our methods, we used logistic regression with robust standard errors to account for clustering by the 20 zones. As described in Table 4 (please see footnotes), we adjusted the analysis for men by study arm (i.e. intervention or control) to account for the fact that there was evidence of an intervention effect amongst older men. We did not include this variable for women as there was no evidence of an effect of the intervention (as presented in Table 2).
